# Fixed-time regulation of spacecraft orbit and attitude coordination with optimal actuation allocation using dual quaternion

**DOI:** 10.3389/frobt.2023.1138115

**Published:** 2023-02-14

**Authors:** Lichao Sun, Yanpei Huang, Haolin Fei, Bo Xiao, Eric M. Yeatman, Allahyar Montazeri, Ziwei Wang

**Affiliations:** ^1^ School of Education, Communication & Society, King’s College London, London, United Kingdom; ^2^ Department of Bioengineering, Imperial College London, London, United Kingdom; ^3^ School of Engineering, Lancaster University, Lancaster, United Kingdom; ^4^ Department of Computing, Imperial College London, London, United Kingdom; ^5^ Department of Electrical and Electronic Engineering, Imperial College London, London, United Kingdom

**Keywords:** dual quaternion, spacecraft control, fixed-time stability, control torque allocation, optimization

## Abstract

On-orbit service spacecraft with redundant actuators need to overcome orbital and attitude coupling when performing proximity maneuvers. In addition, transient/steady-state performance is required to fulfill the user-defined requirements. To these ends, this paper introduces a fixed-time tracking regulation and actuation allocation scheme for redundantly actuated spacecraft. The coupling effect of translational and rotational motions is described by dual quaternion. Based on this, we propose a non-singular fast terminal sliding mode controller to guarantee fixed-time tracking performance in the presence of external disturbances and system uncertainties, where the settling time is only dependent on user-defined control parameters rather than initial values. The unwinding problem caused by the redundancy of dual quaternion is handled by a novel attitude error function. Moreover, optimal quadratic programming is incorporated into null space pseudo-inverse control allocation that ensures the actuation smoothness and never violates the maximum output capability of each actuator. Numerical simulations on a spacecraft platform with symmetric thruster configuration demonstrate the validity of the proposed approach.

## 1 Introduction

Orbit and attitude coordination benefits the motion accuracy and efficiency of spacecraft by controlling orientation and position simultaneously. Several works in non-cooperative target capture (Huang et al., 2006; Zhang et al., 2017) and space teleoperation (Wang et al., 2019a) have demonstrated that these tasks can be performed through separate orientation and position control loops through the corresponding actuators, which ease the stability analysis and control synthesis. However, they might not be directly applied in proximate on-orbit servicing tasks due to the coupling effect between translation and orientation motions. On the one hand, rotation motion driven by reaction flywheels will result in orbital motion and *vice versa*. On the other hand, space load (e.g. space manipulator) motion will impact the pose of the whole system subject to the conservation of angular momentum (Wang et al., 2021).

How to model the coordinated dynamic that involves translation and orientation motions of spacecraft remains challenging for stability analysis and control design. Dual quaternion (DQ) is an alternative to describe the aforementioned coupling effect (Brodsky and Shoham, 1999). With a DQ-based velocity-free controller (Filipe and Tsiotras, 2013), the relative position and attitude were globally asymptotic stable for rigid body motion. The DQ-based modeling also becomes promising in spacecraft formation (Nixon and Shtessel, 2022) and perception (Srivatsan et al., 2016; Reynolds et al., 2020), whereas lacks the consideration of transient-state performance. As an important metric to evaluate transient-state convergence performance, settling time has gradually transformed from control objective to control parameter, thus allowing for more flexible control structures in terms of finite/fixed-time stability (Bhat and Bernstein, 2000; Zhu et al., 2011; Polyakov et al., 2015; Chen et al., 2022; Wang et al., 2022). In terms of faster convergence performance and robustness against disturbances, recent works in (Wang et al., 2020; 2019b) have witnessed that terminal sliding mode control (TSMC) can provide a route for finite-time stability. However, the settling time relies on accurate initial values, which might be limited to practical control implementation. Since noise signal is inevitable for measurement in practice, the resulting settling time tends to enlarge the estimation conservatism.

In addition, spacecraft are generally equipped with redundant sets of actuators in terms of safety. Control allocation plays an important role in over-actuated systems to distribute the control output among the redundant actuators. The fixed, single-gimbal, and double-gimbal thruster configurations were discussed in (Servidia, 2010). In order to improve the torque capacity during maneuvers by determining the initial wheel angular momentum, maximizing the efficiency of torque distribution for low-capacity reaction wheel assemblies was discussed in (Choi et al., 2008). In (Schaub and Lappas, 2009), an optimal torque distribution strategy was developed for reaction wheels to minimize the instantaneous electrical power requirement. In order to allocate the moments of the three axes to the corresponding control surfaces, robust least-square control allocation for unstructured and structured uncertainties was considered with a combination of *H*_2_/*H*_*∞*_ feedback and feedforward control (Cui and Yang, 2011).

Motivated by the above observation, we use DQ to develop a non-singular fixed-time terminal sliding mode control (NFxTSMC) strategy for a 6-degree-of-freedom (DoF) spacecraft with settling time requirement, which can overcome the unwinding problem induced by DQ. Moreover, an optimal null-space based pseudo-inverse (ONSPI) control allocation strategy facilitates alleviating the physical restrictions on actuation characteristics. The main contributions of this paper are presented as follows:

1) The DQ-based control scheme features fixed-time and unwinding-free convergence while handling the coupling between translation and orientation motions. Compared with the previous work (Sun et al., 2022), we have shown the scalability of the proposed control scheme from finite-time to fixed-time stability, where the settling time only relies on the user-defined control parameters rather than initial values.

2) Control allocation strategy in a framework of optimal quadratic programming can address the output constraint of the redundant actuators. Compared with the traditional pseudo-inverse (PI) method, multiple constraints can be incorporated into the cost function that provides superior control allocation performances. Optimal quadratic programming is employed in the null space pseudo inverse control allocation that ensures the actuation smoothness and never violates the maximum output capability of each actuator.

The remainder of this paper is organized as follows. In Section 2, the relative kinematics and dynamics are derived for a class of 6-DoF orbit and attitude coordination spacecraft systems based on DQ. In Section 3, an NFxTSMC is proposed considering external disturbances, system uncertainties, and singularity phenomenon. Furthermore, the control allocation strategy is designed in Section 4. In Section 5, numerical simulations on a platform of spacecraft rendezvous and docking have demonstrated the effectiveness of the proposed control and actuator allocation method, followed by conclusions drawn in Section 6.

## 2 Preliminaries

### 2.1 Dual quaternion

In order to describe the translational and rotational motion simultaneously, we introduce DQ (Brodsky and Shoham, 1999): $\hat{q}\equiv \eta +\epsilon \xi$ , where *η* and *ξ* are the real and dual part, respectively, and *ϵ* the dual operator such that *ϵ* ^2^ = 0, *ϵ* ≠ 0. Here, *η* and *ξ* are both quaternions. In the following, $\hat{\left(\cdot \right)}$ stands for the DQ variable. Taking $\hat{a}=a+\epsilon a^{\prime }$ and $\hat{b}=b+\epsilon b^{\prime }$ as an example, the following operators (Wang and Sun, 2012) are used throughout this paper:

$$\begin{array}{cc} \hat{a}\pm \hat{b} & ≔a\pm b+\epsilon \left(a^{\prime }\pm b^{\prime }\right),\\ {\hat{a}}^{*} & ≔a+\epsilon \left(-a^{\prime }\right),\\ {\hat{a}}^{T} & ≔a^{T}+\epsilon {\left(a^{\prime }\right)}^{T},\\ {\hat{a}}^{-1} & ≔a^{-1}+\epsilon {\left(a^{\prime }\right)}^{-1}, \end{array}$$ (1)

$$\hat{a}\times \hat{b}≔a\times b+\epsilon \left(a\times b^{\prime }+a^{\prime }\times b\right),\;\hat{a}\hat{b}=\hat{a}⊙\hat{b}≔ab+\epsilon a^{\prime }b^{\prime },$$ (2)

$$<\hat{a}|\hat{b}>≔ab+a^{\prime }b^{\prime },\;\left[\hat{a}|\hat{b}\right]≔a^{\text{T}}b^{\prime }+a^{\prime \text{T}}b,$$ (3)

$$\hat{a}\;◦\;\hat{b}≔ab-a^{\prime }b^{\prime }+\epsilon \left(ab^{\prime }+a^{\prime }b+a^{\prime }\times b^{\prime }\right),$$ (4)

$$\hat{a}\leq \hat{b}\;\text{i.f.f.}\;a\leq b\;\text{AND}\;a^{\prime }\leq b^{\prime }.$$ (5)

Given the desired pose ${\hat{q}}_{d}$ , the DQ error can be expressed as:

$${\hat{q}}_{e}={\hat{q}}_{d}^{*}\;◦\;\hat{q}=q_{e}+\epsilon \frac{1}{2}q_{e}\;◦\;p_{e}$$ (6)

where *q*_*e*_ and *p*_*e*_ stand for the quaternion and position errors, respectively. Taking the time derivative of ${\hat{q}}_{e}$ yields:

$${\dot{\hat{q}}}_{e}=\frac{1}{2}{\hat{q}}_{e}\;◦\;{\hat{\omega }}_{e},{\hat{\omega }}_{e}≔\hat{\omega }-{\hat{q}}_{e}^{*}\;◦\;{\hat{\omega }}_{d}\;◦\;{\hat{q}}_{e},$$ (7)

in which $\hat{\omega }$ and ${\hat{\omega }}_{d}$ are the actual and desired velocity motors. Therefore, taking the time derivative of ${\hat{\omega }}_{e}$ , we can obtain the DQ error dynamics (Wang and Sun, 2012):

$$\hat{M}{\dot{\hat{\omega }}}_{e}=\hat{u}+\hat{M}\left[{\hat{\omega }}_{e}\times \left({\hat{q}}_{e}^{*}\;◦\;{\hat{\omega }}_{d}\;◦\;{\hat{q}}_{e}\right)-{\hat{q}}_{e}^{*}\;◦\;{\dot{\hat{\omega }}}_{d}\;◦\;{\hat{q}}_{e}\right]-\hat{\omega }\times \hat{M}\hat{\omega }$$ (8)

where $\hat{M}=m\frac{d}{d\epsilon }I+\epsilon J$ is the dual inertial matrix, *m* the mass, *J* the inertial matrix, *I* the identity matrix with appropriate dimensions. $\hat{u}$ is dual force motor such that

$$\hat{u}={\hat{u}}_{c}+{\hat{u}}_{d}=u_{c}+u_{d}+\epsilon \left(\tau_{c}+\tau_{d}\right),$$ (9)

where *u*_*c*_ and *τ*_*c*_ are the control force and torque to be designed. *u*_*d*_ and *τ*_*d*_ are the external force and torque disturbance.

### 2.2 Necessary lemmas

*Lemma 1* (Bhat and Bernstein, 2000). Consider a class of continuous-time systems

$$\dot{x}=f\left(x,t\right),$$ (10)

where *x* ∈ *U* is the system state and *f* the non-linear function. If there exists a continuously differentiable function $V:U\to R_{+}\cup \left\{0\right\}$ such that $\dot{V}\left(x\right)+\gamma V^{\alpha }\left(x\right)\leq 0$ , with *γ*, *α* ∈ (0, 1), then the equilibrium of the system trajectory (10) is globally finite-time stable and the settling time *T* satisfies $T\leq \frac{V_{0}^{1-\alpha }}{\gamma \left(1-\alpha \right)}$ , in which *V*_0_ is the initial value of *V*.

*Lemma 2* (Polyakov, 2011). The equilibrium of the system trajectory (10) is globally fixed-time stable if there exist positive constants: *α*, *β*, *p*, *q*, *k*, with *pk* < 1 and *qk* > 1, such that

$$\dot{V}\left(x\right)\leq -{\left(\alpha V^{p}\left(x\right)+\beta V^{q}\left(x\right)\right)}^{k},$$ (11)

and the settling time *T* follows:

$$T\leq \frac{1}{\alpha^{k}\left(1-pk\right)}+\frac{1}{\beta^{k}\left(qk-1\right)}.$$ (12)

## 3 Control design and analysis

Considering the 6-DoF trajectory tracking task, the control objective can be stated as follows: design a DQ-based controller ${\hat{u}}_{c}=u_{c}+\epsilon \tau_{c}$ such that the relative error states of a class of spacecraft systems 8) converge within the fixed time, under all time and physically realizable initial conditions. That is, ${\hat{\xi }}_{e}≔{\left(q_{e}\right)}_{v}+\epsilon {\left(q_{e}^{\prime }\right)}_{v}\to {\left[0,0,0\right]}^{\text{T}}+\epsilon {\left[0,0,0\right]}^{\text{T}}$ and ${\hat{\omega }}_{e}\to \hat{0}$ for *t* → *T*, where (⋅)_*v*_ denotes the vector part of quaternion and *T* is the settling time. In the existence of external disturbances and system uncertainties, the error dynamics can be equivalently expressed as:

$$\begin{array}{ll} {\dot{\hat{\xi }}}_{e} & =\hat{\Theta }\left({\hat{q}}_{e}\right){\hat{\omega }}_{e},\\ {\hat{M}}_{0}{\dot{\hat{\omega }}}_{e} & ={\hat{u}}_{c}+{\hat{M}}_{0}\left[{\hat{\omega }}_{e}\times \left({\hat{q}}_{e}^{*}\;◦\;{\hat{\omega }}_{d}\;◦\;{\hat{q}}_{e}\right)-{\hat{q}}_{e}^{*}\;◦\;{\dot{\hat{\omega }}}_{d}\;◦\;{\hat{q}}_{e}\right]\\ & \;-\hat{\omega }\times {\hat{M}}_{0}\hat{\omega }+\hat{\Phi }, \end{array}$$ (13)

where $\hat{\Theta }\left({\hat{q}}_{e}\right)=\frac{1}{2}\left({\hat{\eta }}_{e}I+{\hat{\xi }}_{e}^{\times }\right)$ with ${\dot{\hat{\eta }}}_{e}=-\frac{1}{2}{\hat{\eta }}_{e}^{\text{T}}{\hat{\omega }}_{e}$ . (⋅)^×^ represents the cross product operator. ${\hat{M}}_{0}$ and $\Delta \hat{M}$ the nominal and uncertain part of $\hat{M}$ such that $\hat{M}={\hat{M}}_{0}+\Delta \hat{M}$ and therefore $\hat{\Phi }$ can be written as:

$$\begin{array}{ll} \hat{\Phi } & =-\Delta \hat{M}{\dot{\hat{\omega }}}_{e}+\Delta \hat{M}\left[{\hat{\omega }}_{e}\times \left({\hat{q}}_{e}^{*}\;◦\;{\hat{\omega }}_{d}\;◦\;{\hat{q}}_{e}\right)-{\hat{q}}_{e}^{*}\;◦\;{\dot{\hat{\omega }}}_{d}\;◦\;{\hat{q}}_{e}\right]-\hat{\omega }\\ & \;\times \Delta \hat{M}\hat{\omega }+{\hat{u}}_{d}. \end{array}$$ (14)

In order to stabilize ${\hat{\xi }}_{e}$ and ${\hat{\omega }}_{e}$ within fixed time, traditional sliding mode (Wang et al., 2018) can be modified as dual form:

$${\hat{S}}_{i}=S_{i}+\epsilon S_{i}^{\prime }={\dot{\hat{\xi }}}_{ei}+{\hat{b}}_{i}⊙\text{sig}{\left({\hat{\xi }}_{ei}\right)}^{\alpha },\;i=1,2,3,$$ (15)

where $\hat{S}={\left[{\hat{S}}_{1},{\hat{S}}_{2},{\hat{S}}_{3}\right]}^{\text{T}}$ , $\text{sig}{\left({\hat{\xi }}_{ei}\right)}^{\alpha }=\text{sig}{\left(\xi_{ei}\right)}^{\alpha }+\epsilon \text{sig}{\left(\xi_{ei}^{\prime }\right)}^{\alpha }$ , $\text{sig}\left(\cdot \right)=\text{sgn}\left(\cdot \right){\left|\cdot \right|}^{\alpha }$ , and sgn(⋅) is the sign function. *α* ∈ (0, 1). ${\hat{b}}_{i}=b_{i}+\epsilon b_{i}^{\prime }$ with *b*_*i*_ and $b_{i}^{\prime }$ being positive constants. Based on the terminal sliding mode (15), the resulting TSMC can be then designed as a similar structure in (Dong et al., 2016). However, the implementation of the above algorithm tends to generate excessive control torque since the inclusion of negative exponential terms may lead to singularities.

*Assumption 1*. The real and dual parts of ${\hat{M}}_{0}$ are positive-definite, bounded, and invertible.

*Assumption 2*. $\hat{\Phi }$ is bounded by an unknown dual constant such that $\left\|\hat{\Phi }\|\leq \hat{d}⊙\|\hat{S}\right\|$ .

*Remark 1*. Since the real and dual parts of ${\hat{M}}_{0}$ represent the mass and inertial of the rigid-body spacecraft system, *Assumption 1* can always hold. Since each term in $\hat{\Phi }$ is subject to the measurement range of physical sensors, there exists an upper limitation for $\hat{\Phi }$ that is bounded, which has been also validated in existing literature (Xiao and Yin, 2016).

Inspired by (Wang et al., 2009), a variant of the non-singular sliding mode in dual form is proposed as follows:

$${\hat{S}}_{i}={\dot{\hat{\xi }}}_{ei}+{\hat{\alpha }}_{1i}⊙{\hat{\xi }}_{ei}+{\hat{\alpha }}_{2i}⊙{\hat{S}}_{ai},\;i=1,2,3,$$ (16)

where ${\hat{\alpha }}_{1i}=\alpha_{1i}+\epsilon \alpha_{1i}^{\prime }$ , ${\hat{\alpha }}_{2i}=\alpha_{2i}+\epsilon \alpha_{2i}^{\prime }$ *α*_1*i*_, $\alpha_{1i}^{\prime }$ , *α*_2*i*_, and $\alpha_{2i}^{\prime }$ are positive constants. ${\hat{S}}_{ai}$ is the auxiliary terminal sliding mode:

$$S_{ai}=\left\{\begin{array}{c} \text{sig}{\left(\xi_{ei}\right)}^{p_{1}},\text{if}\;{\bar{S}}_{i}=0\;\text{or}\;{\bar{S}}_{i}\neq 0,\left|\xi_{ei}\right|\geq \delta \;\\ r_{1}\xi_{ei}+r_{2}\text{sig}{\left(\xi_{ei}\right)}^{2},\text{if}\;{\bar{S}}_{i}\neq 0,\left|\xi_{ei}\right|<\delta \; \end{array}\right.$$ (17)

$$S_{ai}^{\prime }=\left\{\begin{array}{c} \text{sig}{\left(\xi_{ei}^{\prime }\right)}^{p_{1}},\text{if}\;{\bar{S}}_{i}^{\prime }=0\;\text{or}\;{\bar{S}}_{i}^{\prime }\neq 0,\left|\xi_{ei}^{\prime }\right|\geq \delta^{\prime }\;\\ r_{1}^{\prime }\xi_{ei}^{\prime }+r_{2}^{\prime }\text{sig}{\left(\xi_{ei}^{\prime }\right)}^{2},\text{if}\;{\bar{S}}_{i}^{\prime }\neq 0,\left|\xi_{ei}^{\prime }\right|<\delta^{\prime }\; \end{array}\right.$$ (18)

$${\hat{\bar{S}}}_{i}={\bar{S}}_{i}+\epsilon {\bar{S}}_{i}^{\prime }={\dot{\hat{\xi }}}_{ei}+{\hat{\alpha }}_{1i}⊙{\hat{\xi }}_{ei}+{\hat{\alpha }}_{2i}⊙\text{sig}{\left({\hat{\xi }}_{ei}\right)}^{p_{1}},$$ (19)

where $r_{1}=\left(2-p_{1}\right)\delta^{p_{1}-1}$ , $r_{1}^{\prime }=\left(2-p_{1}\right)\delta^{\prime p_{1}-1}$ , $r_{2}=\left(p_{1}-1\right)\delta^{p_{1}-2}$ , and $r_{2}^{\prime }=\left(2-p_{1}\right)\delta^{\prime p_{1}-2}$ *δ* and *δ*′ are small positive constants. *p*_1_ ∈ (0.5, 1). On this basis, the NFxTSMC is designed as:

$$\begin{array}{ll} {\hat{u}}_{c}= & -{\hat{M}}_{0}\left[{\hat{\omega }}_{e}\times \left({\hat{q}}_{e}^{*}\;◦\;{\hat{\omega }}_{d}\;◦\;{\hat{q}}_{e}\right)-{\hat{q}}_{e}^{*}\;◦\;{\dot{\hat{\omega }}}_{d}\;◦\;{\hat{q}}_{e}\right]+\hat{\omega }\times {\hat{M}}_{0}\hat{\omega }\\ & \;-\hat{K}\text{sig}\left(\hat{S}\right)-\left({\hat{M}}_{0}\hat{\Theta }{\left({\hat{q}}_{e}\right)}^{-1}\right)\left[{\hat{\alpha }}_{1}⊙{\dot{\hat{\xi }}}_{e}+\dot{\hat{\Theta }}\left({\hat{q}}_{e}\right){\hat{\omega }}_{e}\right.\\ & \;+{\hat{\alpha }}_{2}⊙W\left({\hat{\xi }}_{e}\right)⊙\hat{\Theta }\left({\hat{q}}_{e}\right){\hat{\omega }}_{e}+{\hat{\alpha }}_{3}⊙\text{sig}{\left(\hat{S}\right)}^{p_{2}}\\ & \;+\left.{\hat{\alpha }}_{4}⊙\text{sig}{\left(\hat{S}\right)}^{p_{3}}\right] \end{array}$$ (20)

where ${\hat{\alpha }}_{3}=\alpha_{3}+\epsilon \alpha_{3}^{\prime }$ , ${\hat{\alpha }}_{4}=\alpha_{4}+\epsilon \alpha_{4}^{\prime }$ , and $\hat{K}=K+\epsilon K^{\prime }$ *α*_3_, $\alpha_{3}^{\prime }$ , *α*_4_, $\alpha_{4}^{\prime }$ , *K*, *K*′, and *p*_2_ are positive constants, with *p*_2_ ∈ (0.5, 1) and *p*_3_ > 1. $W\left({\hat{\xi }}_{e}\right)$ is a diagonal matrix whose *i*th entry in the main diagonal is

$$W\left(\xi_{ei}\right)=\left\{\begin{array}{c} p_{1}{\left|\xi_{ei}\right|}^{p_{1}-1},\text{if}\;{\bar{S}}_{i}=0\;\text{or}\;{\bar{S}}_{i}\neq 0,\left|\xi_{ei}\right|\geq \delta \;\\ r_{1}+2r_{2}\left|\xi_{ei}\right|,\text{if}\;{\bar{S}}_{i}\neq 0,\left|\xi_{ei}\right|<\delta \; \end{array}\right.$$ (21)

$$W\left(\xi_{ei}^{\prime }\right)=\left\{\begin{array}{c} p_{1}{\left|\xi_{ei}^{\prime }\right|}^{p_{1}-1},\text{if}\;{\bar{S}}_{i}^{\prime }=0\;\text{or}\;{\bar{S}}_{i}^{\prime }\neq 0,\left|\xi_{ei}^{\prime }\right|\geq \delta^{\prime }\;\\ r_{1}^{\prime }+2r_{2}^{\prime }\left|\xi_{ei}^{\prime }\right|,\text{if}\;{\bar{S}}_{i}^{\prime }\neq 0,\left|\xi_{ei}^{\prime }\right|<\delta^{\prime }\; \end{array}\right.$$ (22)

*Theorem 1*. Given the bounded external disturbances and system uncertainties (14), if the control law (20) is adopted, then the relative DQ and velocity motors of the DQ-based spacecraft system 8) are guaranteed to converge within fixed time *T*_1_ + *T*_2_, which will be provided later.

*Proof*. We will validate the fixed-time performance through the following two steps: i) the system trajectory reaches the sliding mode surface within a fixed time under any initial conditions, and ii) within the fixed time, the system trajectory converges to the equilibrium point alongside the sliding mode surface.

*Step i)*: We adopt the following Lyapunov function candidate: $V_{1}=\frac{1}{2}\left[\hat{S}|{\hat{M}}_{0}\hat{S}\right]$ . Taking the time-derivative of *V*_1_ and substituting (13) into it yields

$$\begin{array}{ll} {\dot{V}}_{1} & =\left[\hat{S}|{\hat{M}}_{0}{\ddot{\hat{\xi }}}_{e}+{\hat{M}}_{0}{\hat{\alpha }}_{1}⊙{\dot{\hat{\xi }}}_{e}+{\hat{M}}_{0}{\hat{\alpha }}_{2}⊙{\dot{\hat{S}}}_{a}\right]\\ & =\left[\hat{S}|{\hat{M}}_{0}\dot{\hat{\Theta }}\left({\hat{q}}_{e}\right){\hat{\omega }}_{e}+{\hat{M}}_{0}{\hat{\alpha }}_{1}⊙{\dot{\hat{\xi }}}_{e}+{\hat{M}}_{0}{\hat{\alpha }}_{2}⊙{\dot{\hat{S}}}_{a}\right.+{\hat{M}}_{0}\hat{\Theta }\left({\hat{q}}_{e}\right)\\ & \;\times {\hat{M}}_{0}^{-1}\left({\hat{u}}_{c}+{\hat{M}}_{0}\left[{\hat{\omega }}_{e}\times \left({\hat{q}}_{e}^{*}\;◦\;{\hat{\omega }}_{d}\;◦\;{\hat{q}}_{e}\right)-{\hat{q}}_{e}^{*}\;◦\;{\dot{\hat{\omega }}}_{d}\;◦\;{\hat{q}}_{e}\right]\right.\\ & \;-\left.\left.\hat{\omega }\times {\hat{M}}_{0}\hat{\omega }+\hat{\Phi }\right)\right]. \end{array}$$ (23)

Substituting the controller (20) into ${\dot{V}}_{1}$ , we can derive

$$\begin{array}{ll} {\dot{V}}_{1}\leq & \left[\hat{S}|{\hat{M}}_{0}{\hat{\alpha }}_{2}⊙{\dot{\hat{S}}}_{a}-{\hat{M}}_{0}\hat{\Theta }\left({\hat{q}}_{e}\right){\hat{M}}_{0}^{-1}\hat{K}\text{sig}\left(\hat{S}\right)\right.\\ & \;-\left.{\hat{M}}_{0}\left({\hat{\alpha }}_{2}⊙W\left({\hat{\xi }}_{e}\right)⊙\hat{\Theta }\left({\hat{q}}_{e}\right){\hat{\omega }}_{e}+{\hat{\alpha }}_{3}⊙\text{sig}{\left(\hat{S}\right)}^{p_{2}}\right.\right.\\ & \;+\left.\left.{\hat{\alpha }}_{4}⊙\text{sig}{\left(\hat{S}\right)}^{p_{3}}\right)+{\hat{M}}_{0}\hat{\Theta }\left({\hat{q}}_{e}\right){\hat{M}}_{0}^{-1}⊙\hat{d}⊙\|\hat{S}\|\right]\\ & \;\leq \left[\hat{S}|-{\hat{M}}_{0}{\hat{\alpha }}_{3}⊙\text{sig}{\left(\hat{S}\right)}^{p_{2}}-{\hat{M}}_{0}{\hat{\alpha }}_{4}⊙\text{sig}{\left(\hat{S}\right)}^{p_{3}}\right]\\ & \;\leq -\underline{\mu }\left(\alpha_{3}{\left\|S_{i}\right\|}^{p_{2}+1}+\alpha_{3}^{\prime }{\left\|S_{i}^{\prime }\right\|}^{p_{2}+1}\right.\\ & \;+\left.\alpha_{4}{\left\|S_{i}\right\|}^{p_{3}+1}+\alpha_{4}^{\prime }{\left\|S_{i}^{\prime }\right\|}^{p_{3}+1}\right)\\ & \;\leq -\mu_{1}V_{1}^{\frac{p_{2}+1}{2}}-\mu_{2}V_{1}^{\frac{p_{3}+1}{2}} \end{array}$$ (24)

where $\underline{\mu }=\text{min}\left\{\sigma_{\text{min}}\left(J\right),m\right\}$ , *σ*_min_(⋅) is the minimum eigenvalue, $\mu_{1}=2^{\frac{p_{2}+1}{2}}\underline{\mu }\text{min}\left\{\alpha_{3},\alpha_{3}^{\prime }\right\}$ , and $\mu_{2}=2^{\frac{p_{3}+1}{2}}\underline{\mu }\text{min}\left\{\alpha_{4},\alpha_{4}^{\prime }\right\}$ . Thus, using the *Lemma* 2, the state trajectory will reach the sliding mode within fixed time *T*_1_, where the settling time can be expressed as:

$$T_{1}\leq \frac{2}{\mu_{1}\left(1-p_{2}\right)}+\frac{2}{\mu_{2}\left(p_{3}-1\right)}.$$ (25)

It therefore implies that ${\hat{S}}_{i}=\hat{0}$ for *i* = 1, 2, 3 after *T*_1_. It is worth pointing out that the coefficients in the upper bound of *T*_1_ are only determined by the user-defined parameters, which are independent of initial conditions. When *t* > *T*_1_, one can obtain

$${\dot{\hat{\xi }}}_{ei}=-{\hat{\alpha }}_{1i}⊙{\hat{\xi }}_{ei}-{\hat{\alpha }}_{2i}⊙{\hat{S}}_{ai}.$$ (26)

*Step ii)*: Consider the following Lyapunov function: $V_{2}=\frac{1}{2}<{\hat{\xi }}_{e}|{\hat{\xi }}_{e}>$ . Taking the time derivation of *V*_2_, we have

$$\begin{array}{ll} {\dot{V}}_{2} & =-\overset{3}{\underset{i=1}{\sum }}\left(\alpha_{1i}{\left|\xi_{ei}\right|}^{2}+\alpha_{1i}^{\prime }{\left|\xi_{ei}^{\prime }\right|}^{2}\right.\\ & \;+\left.\alpha_{2i}{\left|\xi_{ei}\right|}^{p_{1}+1}+\alpha_{2i}^{\prime }{\left|\xi_{ei}^{\prime }\right|}^{p_{1}+1}\right)\leq -\mu_{3}V_{2}^{\frac{1+p_{1}}{2}}, \end{array}$$ (27)

where $\mu_{3}=\text{min}\left\{\alpha_{1i},\alpha_{1i}^{\prime },\alpha_{2i},\alpha_{2i}^{\prime }\right\}$ for *i* = 1, 2, 3. According to the *Lemma* 1, the system trajectory on the sliding mode is guaranteed to converge to equilibrium within finite time *T*_2_, namely ${\hat{\xi }}_{e}\to {\left[0,0,0\right]}^{\text{T}}+\epsilon {\left[0,0,0\right]}^{\text{T}}$ , ${\hat{\omega }}_{e}\to \hat{0}$ , and $T_{2}\leq 2V_{2}^{\frac{1-p_{1}}{2}}\left(T_{1}\right)/\mu_{3}\left(1-p_{1}\right)$ .

*Remark 2*. The system trajectory will enter the asymptotic sliding mode from the terminal sliding one when the sliding mode variables in (20) approach zero. This mechanism ensures singularity-free performance in the convergence procedure. In terms of the parameter selection rule, the error states will converge within the fixed settling time if larger *μ*_1_, *μ*_2_, *μ*_3_, and smaller *p*_1_ and *p*_2_ are chosen. *K* and *K*′ are suggested to be large enough for robustness against external disturbances and system uncertainties.

*Remark 3*. Traditional TSMC generates negative exponential terms of state variables and can therefore lead to singularities. In contrast, the proposed controller (20) is non-singular due to the following facts: state variables are not small enough to cause singularity for $\hat{\bar{S}}\neq \hat{0}$ ; in terms of $\hat{\bar{S}}=\hat{0}$ , the dual controller can be transformed as:

$$\begin{array}{ll} {\hat{u}}_{c} & =-{\hat{M}}_{0}\left[{\hat{\omega }}_{e}\times \left({\hat{q}}_{e}^{*}\;◦\;{\hat{\omega }}_{d}\;◦\;{\hat{q}}_{e}\right)-{\hat{q}}_{e}^{*}\;◦\;{\dot{\hat{\omega }}}_{d}\;◦\;{\hat{q}}_{e}\right]+\hat{\omega }\times {\hat{M}}_{0}\hat{\omega }\\ & \;-\hat{K}\text{sig}\left(\hat{S}\right)-\left({\hat{M}}_{0}\hat{\Theta }{\left({\hat{q}}_{e}\right)}^{-1}\right)\left[{\hat{\alpha }}_{1}⊙{\dot{\hat{\xi }}}_{e}+\dot{\hat{\Theta }}\left({\hat{q}}_{e}\right){\hat{\omega }}_{e}+p_{1}\right.\\ & \;\times \left.{\hat{\alpha }}_{2}⊙\text{sig}{\left({\hat{\xi }}_{e}\right)}^{2p_{1}-1}+{\hat{\alpha }}_{3}⊙\text{sig}{\left(\hat{S}\right)}^{p_{2}}+{\hat{\alpha }}_{4}⊙\text{sig}{\left(\hat{S}\right)}^{p_{3}}\right]. \end{array}$$ (28)

Therefore, the singularity phenomenon will not occur if *p*_1_ ∈ (0.5, 1).

The double value of quaternions results in the unwinding problem of attitude slewing, thereby degrading the global stability of the closed-loop system (Zheng et al., 2017). Here, an attitude error function is employed to overcome the unwinding problem as follows:

$$\phi =2\left(\lambda_{1}+\lambda_{2}q_{e0}^{2}-\text{exp}\left(\frac{q_{e0}^{2}}{\sqrt{q_{e0}^{2}+\mu }}\right)\right),$$ (29)

$$e_{r}=\left(\text{exp}\left(\frac{q_{e0}^{2}}{\sqrt{q_{e0}^{2}+\mu }}\right)\frac{q_{e0}\left(\left|q_{e0}\right|+2\mu \right)}{{\left(\left|q_{e0}\right|+\mu \right)}^{2}}\right){\left(q_{e}\right)}_{v}.$$ (30)

where *λ*_1_, *λ*_2_, and *μ* are positive constants. *q*_*e*0_ is the real part of *q*_*e*_. The proposed attitude error vector is obviously continuous and bounded with *θ* ∈ [−*π*, *π*], which guarantees the response rate of the attitude error vector and the continuity of the attitude error function simultaneously. Thus, the anti-unwinding NFxTSMC can be obtained by replacing the original attitude error function and vector by (29)-(30). It can be derived that the anti-unwinding state is updated as ${\hat{\xi }}_{e}^{*}=e_{r}+\epsilon \left(e_{r}\;◦\;p_{e}\right)$ .

## 4 Optimal control torque allocation strategy

To improve reliability and safety, redundant actuators are often equipped with spacecraft systems to provide corresponding forces and torques. Inspired by (Gersh and Peck, 2009), consider the following constraint condition in dual framework

$${\hat{u}}_{c}\left(t\right)=\hat{D}⊙{\hat{u}}_{a}\left(t\right)$$ (31)

where ${\hat{u}}_{a}$ denotes the actuation output, and $\hat{D}$ the control allocation matrix. Without consideration of the actuator installment faults, the PI control allocation strategy can be ideally presented as follows

$${\hat{u}}_{a}\left(t\right)={\hat{D}}^{†}⊙{\hat{u}}_{c}\left(t\right).$$ (32)

where ${\hat{D}}^{†}={\hat{D}}^{\text{T}}⊙{\left(\hat{D}⊙{\hat{D}}^{\text{T}}\right)}^{-1}$ is the Moore–Penrose inverse of $\hat{D}$ . The linear mapping between ${\hat{u}}_{a}\left(t\right)$ and ${\hat{u}}_{c}\left(t\right)$ is presented through the PI control allocation. However, the solution given by (32) may not satisfy the practical thruster range with the limitation of the thruster configuration (Tang et al., 2011). Thus, the optimal solution can be improved by employing the null space of the control allocation matrix

$${\hat{u}}_{a}\left(t\right)={\hat{D}}^{†}⊙{\hat{u}}_{c}\left(t\right)+\hat{\zeta }\left(t\right)$$ (33)

where $\hat{D}⊙\hat{\zeta }\left(t\right)=\hat{0}$ , namely $\text{Null}\left(\hat{D}\right)=\left\{\hat{\zeta }\left(t\right)|\hat{D}⊙\hat{\zeta }\left(t\right)=\hat{0}\right\}$ . Thus, the thruster output can be adjusted to the available range with the proper choice of $\hat{\zeta }\left(t\right)$ . Furthermore, $\hat{\zeta }$ can be expressed as: $\hat{\zeta }\left(t\right)=\hat{\chi }\left(t\right)⊙\hat{\Gamma }$ , where $\hat{\chi }\left(t\right)=\left[{\hat{\chi }}_{1}\left(t\right),{\hat{\chi }}_{2}\left(t\right),\ldots ,{\hat{\chi }}_{n-6}\left(t\right)\right]$ is the basic solution of null space, and $\hat{\Gamma }={\left[{\hat{\Gamma }}_{1},{\hat{\Gamma }}_{2},\ldots ,{\hat{\Gamma }}_{n-6}\right]}^{\text{T}}$ is the undetermined coefficient. Considering the smoothness of the actuator outputs (Hu et al., 2014; Li et al., 2015; Bai et al., 2022), the ONSPI control allocation can be described as an optimization problem

$$\begin{array}{ll} \underset{\hat{\zeta }\left(t\right)}{\text{min}}J\left(\hat{\zeta }\left(t\right)\right) & =\frac{1}{2}\left[\hat{\zeta }\left(t\right)|\hat{L}⊙\hat{\zeta }\left(t\right)\right]\\ & \;+\frac{1}{2}\left[{\hat{u}}_{a}\left(t\right)-{\hat{u}}_{a}\left(t-1\right)|\hat{Q}⊙\left({\hat{u}}_{a}\left(t\right)-{\hat{u}}_{a}\left(t-1\right)\right)\right]\\ & \;+\frac{1}{2}\left[{\hat{u}}_{a}\left(t\right)-{\hat{u}}_{a}\left(t-2\right)|\hat{R}⊙\left({\hat{u}}_{a}\left(t\right)-{\hat{u}}_{a}\left(t-2\right)\right)\right]\\ & \;\text{s.t.}\;{\hat{G}}_{1}\left(t\right)\leq \hat{\zeta }\left(t\right)\leq {\hat{G}}_{2}\left(t\right) \end{array}$$ (34)

where ${\hat{G}}_{1}\left(t\right)={\hat{u}}_{\text{min}}\left(t\right)-{\hat{D}}^{†}⊙{\hat{u}}_{c}\left(t\right)$ , ${\hat{G}}_{2}\left(t\right)={\hat{u}}_{\text{max}}\left(t\right)-{\hat{D}}^{†}⊙{\hat{u}}_{c}\left(t\right)$ , and ${\hat{u}}_{\text{min}}\left(t\right)$ and ${\hat{u}}_{\text{max}}\left(t\right)$ are the known minimum and maximum outputs of the actuators, respectively. $\hat{L}$ , $\hat{Q}$ , and $\hat{R}$ are positive and diagonal weighting matrices with appropriate dimensions, respectively. With the Lagrange multipliers ${\hat{\varphi }}_{1}\leq \hat{0}$ and ${\hat{\varphi }}_{2}\geq \hat{0}$ , we can construct the Lagrangian function corresponding to the constrained optimization problem (34)

$$\begin{array}{ll} L\left(\hat{\zeta }\left(t\right),{\hat{\varphi }}_{1},{\hat{\varphi }}_{2}\right) & =\frac{1}{2}\left[\hat{\zeta }\left(t\right)|\hat{L}⊙\hat{\zeta }\left(t\right)\right]+\;\frac{1}{2}\left[{\hat{u}}_{a}\left(t\right)-{\hat{u}}_{a}\left(t-1\right)|\right.\\ & \;\times \left.\hat{Q}⊙\left({\hat{u}}_{a}\left(t\right)-{\hat{u}}_{a}\left(t-1\right)\right)\right]+\frac{1}{2}\left[{\hat{u}}_{a}\left(t\right)-{\hat{u}}_{a}\right.\\ & \;\times \left.\left(t-2\right)|\hat{R}⊙\left({\hat{u}}_{a}\left(t\right)-{\hat{u}}_{a}\left(t-2\right)\right)\right]\\ & \;+\left[{\hat{\varphi }}_{1}|\left(\hat{\zeta }\left(t\right)-{\hat{G}}_{1}\left(t\right)\right)\right]\\ & \;+\left[{\hat{\varphi }}_{2}|\left(\hat{\zeta }\left(t\right)-{\hat{G}}_{2}\left(t\right)\right)\right], \end{array}$$ (35)

leading to the Karush–Kuhn–Tucker (KKT) condition as follows:

$$\left\{\begin{array}{c} {\hat{G}}_{1}\left(t\right)\leq \hat{\zeta }\left(t\right)\leq {\hat{G}}_{2}\left(t\right),\;\\ {\hat{\varphi }}_{1}\leq \hat{0},\;{\hat{\varphi }}_{2}\geq \hat{0},\;\\ \nabla_{\hat{\zeta }\left(t\right)}L\left(\hat{\zeta }\left(t\right),{\hat{\varphi }}_{1},{\hat{\varphi }}_{2}\right)=\hat{0}\;\\ \left[{\hat{\varphi }}_{1}|\left(\hat{\zeta }\left(t\right)-{\hat{G}}_{1}\left(t\right)\right)\right]=0,\;\left[{\hat{\varphi }}_{2}|\left(\hat{\zeta }\left(t\right)-{\hat{G}}_{2}\left(t\right)\right)\right]=0,\; \end{array}\right.$$ (36)

where one of the feasible solutions can be represented as:

$$\hat{\zeta }\left(t\right)=\hat{A}⊙\left[\hat{Q}⊙\hat{\zeta }\left(t-1\right)+\hat{R}⊙\hat{\zeta }\left(t-2\right)+\hat{B}\right]$$ (37)

where $\hat{A}={\left(\hat{L}+\hat{Q}+\hat{R}\right)}^{-1}$ and $\hat{B}=\hat{Q}⊙{\hat{D}}^{†}⊙{\hat{u}}_{c}\left(t-1\right)+\hat{R}⊙{\hat{D}}^{†}⊙{\hat{u}}_{c}\left(t-2\right)-\left(\hat{Q}+\hat{R}\right)⊙{\hat{D}}^{†}⊙{\hat{u}}_{c}\left(t\right)$ .

## 5 Simulation results

To verify the effectiveness of the proposed NFxTSMC (20), simulations have been carried out using the rigid-body spacecraft system governed by (8). The TSMC (Dong et al., 2016) and non-singular fast terminal sliding mode control (NFTSMC) (Sun et al., 2022) are carried out for comparison, where the identical initial values are selected for different controllers. In particular, the control parameters are chosen for purpose of the same settling time. To eliminate the chattering phenomenon caused by the sign function in (20), the hyperbolic tangent function is used as a substitution.

It is assumed that the spacecraft moves in a circular orbit with a height of 42240 km. The initial relative attitude and position of the spacecraft are chosen as *ρ*_*e*_(0) = [−20,−10,−10]^T^m, *q*_*e*_(0) = [0.6245,0.5,0.5196,0.3]^T^, *ρ*_*ed*_ = [0,0,0]^T^m, *q*_*ed*_ = [1,0,0,0]^T^, *ω*_*e*_(0) = [0,0,0]^T^rad/s. The external disturbance force and torque are *u*_*d*_ = 10^–2^ × [6 + 3 sin(0.6*t*),5 + 4 sin(0.9*t*),4 + sin(0.5*t*)]^T^N and *τ*_*d*_ = 10^–5^ × [2 + 50 sin(0.8*t*),3 + 30 sin(0.5*t*),1 + 70 sin(0.3*t*)]^T^Nm. The nominal mass and inertia are *m*_0_ = 100 kg and *J*_0_ = diag{18, 18, 24}kgm^2^ while the actual ones are *m* = 95 kg and *J* = diag{17, 17, 22}kgm^2^. The control parameters are set as: *α* = 0.67, *p*_1_ = 0.8, *p*_2_ = 0.9, *p*_3_ = 1.2, $\hat{\delta }=0.5+\epsilon 0.0001$ , $\hat{K}=1.2+\epsilon 1.2$ , ${\hat{\alpha }}_{1}={\hat{\alpha }}_{2}=\hat{b}=0.2+\epsilon 0.2$ , ${\hat{\alpha }}_{3}={\hat{\alpha }}_{4}=20+\epsilon 20$ . $\hat{L}=200I_{10}+\epsilon 200I_{10}$ , $\hat{Q}=10I_{10}+\epsilon 10I_{10}$ , $\hat{R}=20I_{10}+\epsilon 20I_{10}$ , and $I_{10}\in R^{10\times 10}$ is the inertial matrix.

**FIGURE 1:**
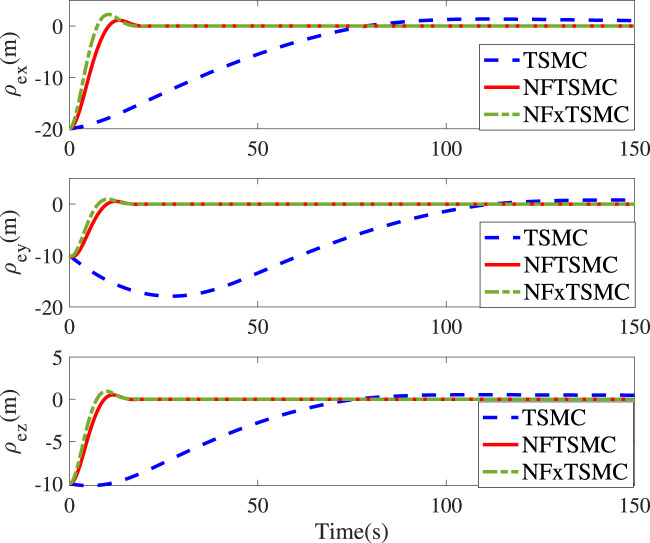
Time responses of ρ_*e*_ for different controllers.

**FIGURE 2:**
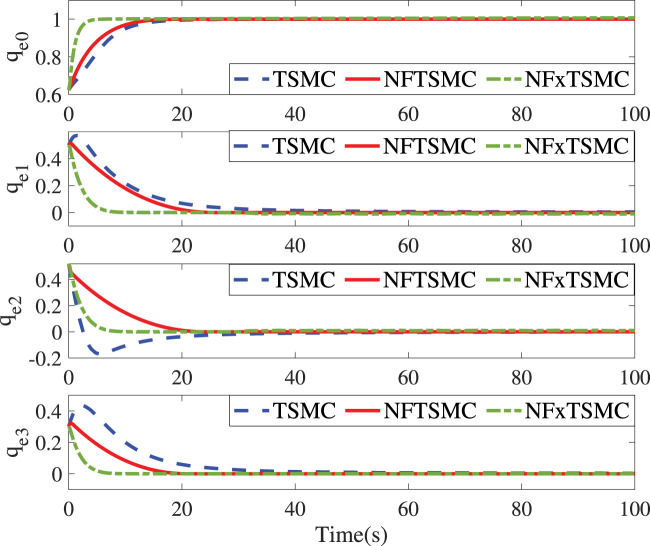
Time responses of *q*_*e*_ for different controllers.

Figure 1; Figure 2 represent the time responses of relative position and attitude errors under the effect of the three controllers. Under the same initial values, the relative position errors driven by the TSMC and NFTSMC converge within 78s and 22s, respectively. In contrast, the proposed NFxTSMC realizes the fastest convergence performance (19s) due to the fact that the convergence rate and accuracy are simultaneously considered in the sliding mode and controller design. In Figure 2, the relative quaternion errors in the proposed controller converge with less overshoot and a higher convergence rate compared with other methods. It is noted that the singularity phenomenon is eliminated in the proposed controller. In order to further validate the fixed-time performance provided by NFxTSMC, we introduce different initial values as follows:

**Case1**: *ρ*_*e*_(0) = [−250,−150,100]^T^m, *q*_*e*_(0) = [0.7,0.4,0.3,0.51]^T^, and *ω*_*e*_(0) = [0,0,0]^T^rad/s;

**Case2**: *ρ*_*e*_(0) = [2500,−1500,−1000]^T^m, *q*_*e*_(0) = [0.3,0.4,0.3,0.81]^T^, and *ω*_*e*_(0) = [0.2,−0.3,0.5]^T^rad/s.

**FIGURE 3:**
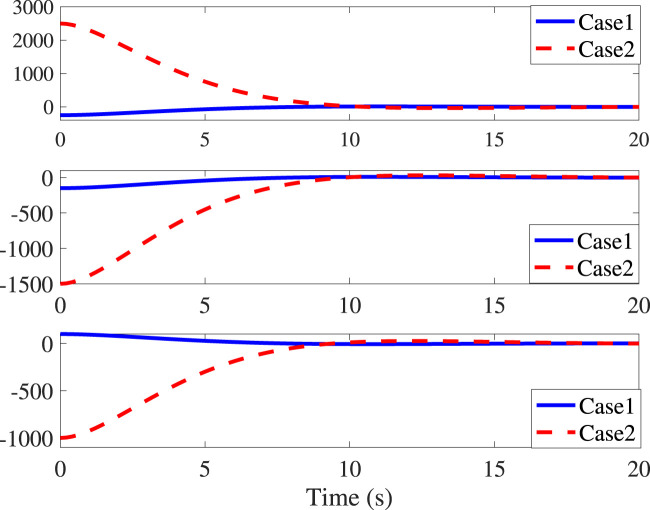
Time responses of ρ_*e*_ in Cases 1 and 2.

**FIGURE 4:**
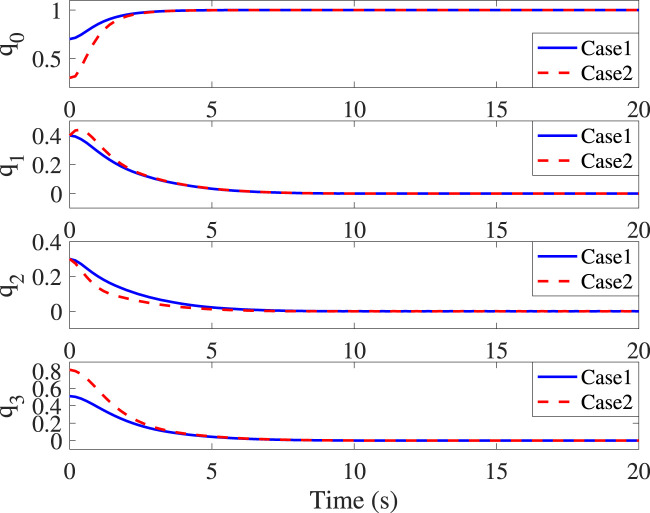
Time responses of *q*_*e*_ in Cases 1 and 2.

The control parameters and objectives in Cases 1 and 2 remain identical to the above settings. It can be seen from Figure 3 that initial values do not impact the settling time (10s) and the corresponding translational convergence performance, while Figure 4 demonstrates the identical settling time of attitude variables under different initial conditions. It, therefore, validates that the proposed NFxTSMC ensures fixed-time stability without the need for exact initial values. Moreover, Figures 1–4 indicate NFxTSMC can realize robust transient-state performance with less overshoot.

**FIGURE 5:**
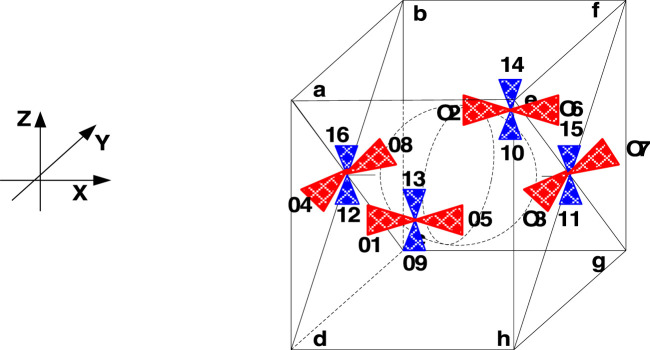
Symmetric thruster configuration (STC).

The symmetric thruster configuration (Sun et al., 2022) (see Figure 5) is employed to test the proposed ONSPI control allocation scheme. Attitude and orbit control corresponding to thrusters with respect to *x*, *y*, and *z* axles are summarized in Table  1.

**TABLE 1:** Thruster configuration corresponding to STC.

	+X	-X	+Y	-Y	+Z	-Z
Attitude Control	#9 + #14	# 10 + #13	#11 + #16	#12 + #15	#1 + #6	#2 + #5
Orbit Control	#1 + #2	#5 + #6	#3 + #4	#7 + #8	#9 + #10 + #11 +#12	#13 + #14 + #15 +#16

**FIGURE 6:**
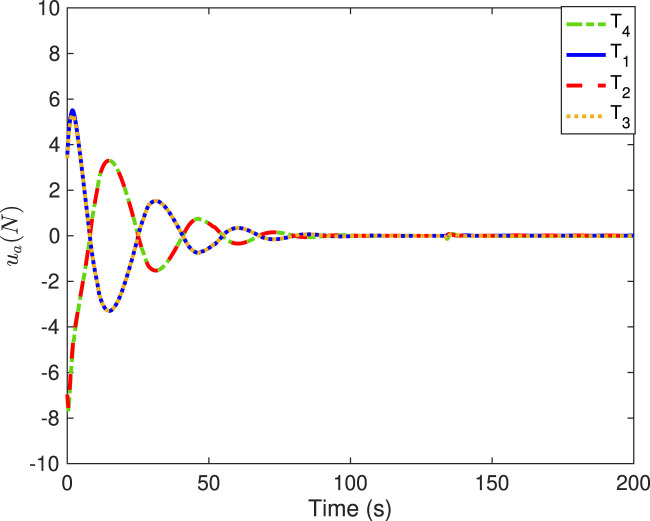
Thruster forces of #1-#4 in the STC.

**FIGURE 7:**
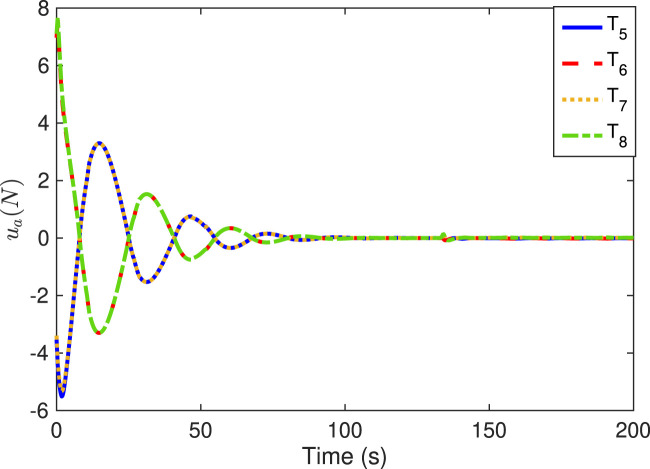
Thruster forces of #5-#8 in the STC.

**FIGURE 8:**
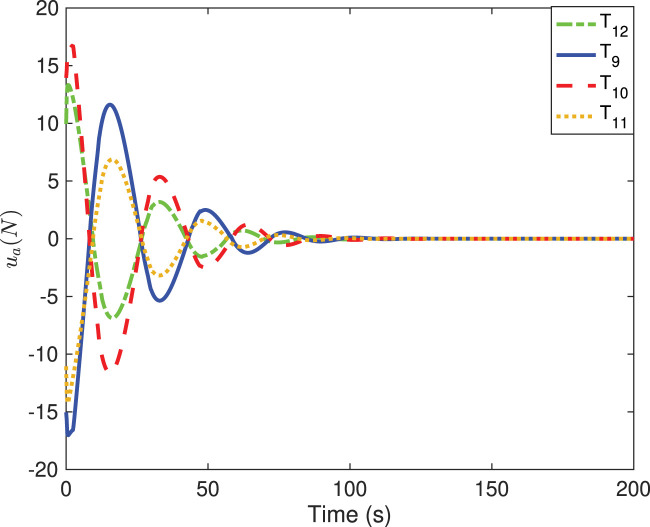
Thruster forces of #9-#12 in the STC.

**FIGURE 9:**
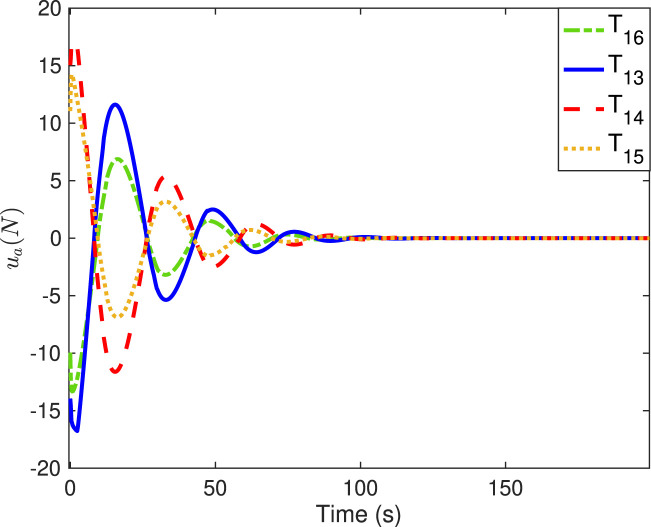
Thruster forces of #13-#16 in the STC.

Figures 6–9 show the practical thruster output in the STC, where *T*_*i*_ corresponds to the *i*th thruster for *i* = 1, 2, … , 16. As observed, pair-mounted actuators can provide symmetrical thrusts. The feasible solution can be found in the pseudo-inverse method within the thrust limitation (20 N) in the STC, where the negative values can also be offered by the thruster from the other direction. Compared with the conventional PI method, the ONSPI method can satisfy control allocation requirements despite control force limitations. Similarly, it is demonstrated in Figures 6–9 that the ONSPI approach can generate smooth actuator output and provide closed-looped stability against external disturbances.

## 6 Conclusion

In this paper, we extend the result in (Sun et al., 2022) from finite-time to fixed-time stability, where the settling time of the spacecraft system is only dependent on user-defined control parameters rather than initial values. The proposed non-singular fixed-time control law provides a more accurate and robust estimation of the settling time compared with finite-time control. Thus, it will benefit the application scenarios with measurement errors and unknown environments. Meanwhile, we demonstrate the scalability of the developed non-singular fixed-time control framework which facilitates alleviating the unwinding problem. Furthermore, the disadvantages of the traditional pseudo-inverse method are eliminated by the optimal quadratic programming, which ensures that all the practical actuator outputs are subject to limitation. Finally, numerical simulations to evaluate the overall performances for non-singularity, fast tracking, high accuracy, uncertainty resistance, and fixed-time stability have verified the effectiveness of the proposed method. The actuator faults and fault-tolerant coordinated controller will be considered in future work.

## Data Availability

The original contributions presented in the study are included in the article/supplementary material, further inquiries can be directed to the corresponding author.
